# The healthy human gut can take it all: vancomycin-variable, linezolid-resistant strains and specific bacteriocin-species interplay in *Enterococcus* spp.

**DOI:** 10.1128/aem.01699-24

**Published:** 2024-12-19

**Authors:** Ana C. Almeida-Santos, Bárbara Duarte, Ana P. Tedim, Maria J. Teixeira, Joana C. Prata, Rui M. S. Azevedo, Carla Novais, Luísa Peixe, Ana R. Freitas

**Affiliations:** 1 UCIBIO, Unidade de Ciências Biomoleculares Aplicadas, Faculdade de Farmácia, Universidade do Porto 26706 , Porto, Portugal; 2 Laboratório Associado i4HB, Instituto para a Saúde e a Bioeconomia, Faculdade de Farmácia, Universidade do Porto 26706 , Porto, Portugal; 3 Grupo de Investigación Biomédica en Sepsis – BioSepsis, Instituto de Investigación Biomédica de Salamanca (IBSAL) 542009 , Salamanca, Spain; 4 Centro de Investigación Biomédica en Red en Enfermedades Respiratorias (CIBERES, CB22/06/00035), Instituto de Salud Carlos III 91837 , Madrid, Spain; 5 Laboratório Associado i4HB, Instituto para a Saúde e a Bioeconomia, Instituto Universitário de Ciências da Saúde, IUCS-CESPU 166433 , Gandra, Portugal; 6 UCIBIO, Unidade de Ciências Biomoleculares Aplicadas, Instituto Universitário de Ciências da Saúde (1H-TOXRUN, IUCS-CESPU) 622447 , Gandra, Portugal; Norwegian University of Life Sciences, Ås, Norway

**Keywords:** *Enterococcus *spp., healthy humans, antimicrobial resistance, *Enterococcus lactis*, bacteriocins, linezolid resistance, VVE

## Abstract

*Enterococcus* spp. are opportunistic human pathogens colonizing the human gut and a significant reservoir for the continuous adaptation of hospital clones. However, studies on the features of enterococci species co-colonizing healthy individuals are scarce. We investigated the prevalence, antibiotic resistance, and bacteriocin profiles of *Enterococcus* species in fecal samples from healthy adults in Portugal using culture-based methods, WGS, and bacteriocin inhibition assays. Results were compared with data from a 2001 study in the same region. *Enterococcus* spp. (*n* = 315; 24% MDR) were recovered from all volunteers. *Enterococcus lactis* was the prevalent species (75%), followed by *Enterococcus faecalis* (65%) and *Enterococcus faecium* (47%). *E. lactis* prevalence increased 2.5-fold since 2001. Linezolid resistance genes (*optrA/poxtA*) were detected in *E. faecium* and *Enterococcus thailandicus* isolates, while a vancomycin-variable *E. faecium* was also identified. Virulence and plasmid profiles were diverse across species, with evidence of exchange of virulence markers and plasmid replicons between *E. faecium* and *E. lactis*. Bacteriocin gene repertoires were extensive and species-specific. Higher numbers of bacteriocin genes were associated with stronger inhibition profiles, and 25% of *E. faecium* and *E. lactis* isolates were capable of inhibiting relevant VRE clones. This study unveils the co-occurrence and ecological dynamics of *Enterococcus* species in the healthy human gut, reinforcing its role as a reservoir for key antibiotic resistance genes and potentially pathogenic strains. The shift toward *E. lactis* prevalence and the detection of linezolid resistance genes in healthy individuals underscore the need for ongoing surveillance of the gut microbiome to guide public health strategies and antibiotic stewardship efforts.

**IMPORTANCE:**

This study highlights the role of *Enterococcus* species in the healthy human gut, revealing important insights into their prevalence and antibiotic resistance. It emphasizes that the human gut serves as a significant reservoir for antibiotic-resistant strains and shows a notable increase and prevalence of *Enterococcus lactis,* which has been underappreciated due to identification challenges. The research also underscores the bacteriocins’ role in microbial competition, where commensal strains inhibit clinical VRE, potentially aiding the restoration of the gut microbiota, after antibiotic treatment. The findings accentuate the need for ongoing surveillance to track changes in gut bacteria, especially with the emergence of resistance genes to last resort antibiotics. Such monitoring is crucial for shaping public health strategies and managing the growing threat of antibiotic-resistant infections. Profiling bacteriocins at the species and strain level can identify ecological adaptation factors and inform strategies to target high-risk clones.

## INTRODUCTION

Enterococci are remarkably adaptable and ubiquitous in nature, thriving in diverse environments such as the gastrointestinal tract of several animals. In humans, they predominately colonize the ileum and colon, constituting up to 1% of the adult intestinal microbiota (1). *Enterococcus faecium* and *Enterococcus faecalis* are for long recognized as the dominant enterococci species in human fecal content (1, 2). Concomitantly, subpopulations of these commensal opportunistic species are frequently involved in urinary tract, endocarditis, and bloodstream infections, producing hospital outbreaks (1, 3). Their proportion increases in the microbiota of elderly and hospitalized individuals (4) as well as in people from low-income countries with poor sanitation (5). As so, the acquisition of antibiotic resistance traits by pathogenic clones of *E. faecium* and *E. faecalis* is clinically significant.

Within *E. faecium*, current phylogenomic analysis divides *E. faecium* into clade A1 (mostly clinical isolates) and A2 (animal- and community-associated isolates). The former clade B was recognized as a separate species, *Enterococcus lactis* (6). Clade A1 is known for its hospital-associated strains (HA-Efm) that exhibit increased antimicrobial resistance (AMR) and virulence determinants, enabling them to thrive in hospital environments (3, 7). Although we can assume that *E. lactis* predominantly inhabits the human gut by past genomic studies referring to clade B (4, 8), we lack detailed data on the prevalence and co-existence of *E. faecium* and *E. lactis* in our gastrointestinal tract (9, 10). Despite *E. lactis* being mostly associated with the community, they can also cause infections in immunocompromised individuals (7, 11, 12).

Several studies document the frequency of resistant enterococci in clinical specimens and fecal samples from hospitalized patients, but only a few have addressed the occurrence of antibiotic-resistant *Enterococcus* spp. in healthy adults, mostly outside of Europe. Some of the most recent studies evaluating the fecal carriage of enterococci in healthy adults from Europe include isolates from the 2000s in Portugal and Spain (2, 4, 8), as well as those collected during 2014–2015 in the Netherlands (13). When available, such studies reported the prevalence of *E. faecium* (73%) over *E. faecalis* (27%) (8) or a similar prevalence of these species (*E. faecium* 47%; *E. faecalis* 43%) (4). Other recent studies have focused specifically on isolating linezolid-resistant enterococci (14). Additionally, few studies have thoroughly analyzed the co-occurrence of different enterococci species or provided detailed genomic characterization of distinct strains within the same sample (15, 16).

We aimed to assess the fecal carriage rate of *Enterococcus* spp. in contemporary samples obtained from healthy Portuguese individuals and compared the results with samples collected 20 years earlier. This study is particularly important as it provides one of the first comprehensive analyses of *E. lactis* alongside other *Enterococcus* species in healthy volunteers. Our comprehensive strain-level analysis of AMR, virulence, and bacteriocin profiles will enhance our understanding of the complex interactions and evolutionary dynamics within the gut microbiota.

## MATERIALS AND METHODS

### Study design and participant recruitment

Fresh stool specimens were collected between February and July of 2022 from healthy individuals living in the Porto district (18 cities), Portugal. Participants were provided with stool collection kits and instructions on how to collect a sample of stool. Fresh stool samples (*n* = 51; one per individual) were collected, refrigerated until transport, and transported to the laboratory within 3 h and either processed immediately or frozen at −20°C until use. Volunteers aged 18 and above, who had not taken antibiotics in the 3 months before collection, were eligible. Additionally, we conducted a comparison of enterococci species distribution and antibiotic susceptibility spanning 2 decades by including isolates obtained in 2001 from human fecal swabs of individuals living in the same region (2). To ensure unbiased comparisons, enterococcal species and antibiotic resistance rates were compared by including only the samples (*n* = 59; 76 isolates) that were recovered from antibiotic-free media.

### Sample processing, species identification, and antimicrobial susceptibility testing

Samples were weighed (2 g), pre-enriched in brain-heart infusion (BHI) broth (18 h/37°C) at 1:10 ratio with and without three antibiotics (ampicillin 16 µg/mL, vancomycin 6 µg/mL, or linezolid 4 µg/mL) and plated (100 µL) onto Slanetz-Bartley agar media (48 h/37°C) with and without the same antibiotics. For each sample-plate pair, one colony of each morphological type (typical of enterococci) was selected, subcultured onto BHI agar, and identified to the species level by PCR assays targeting species-specific genes: *glu*P in the case of *E. faecium* and *E. lactis* (11), and *sod*A for *E. faecalis*, *Enterococcus gallinarum, Enterococcus casseliflavus, Enterococcus raffinosus, Enterococcus durans,* and *Enterococcus hirae* (17). Isolates yielding negative results were identified by MALDI-TOF mass spectrometry (Bruker MALDI Biotyper). Antimicrobial susceptibility testing was performed by disk diffusion against 10 antibiotics (ampicillin, vancomycin, linezolid, erythromycin, tetracycline, ciprofloxacin, high-level streptomycin, high-level gentamicin, quinupristin-dalfopristin, and chloramphenicol) (Oxoid, Basingstoke, UK) according to EUCAST (18) or CLSI (19) guidelines. Resistance rates are presented by sample, rather than by isolates. Minimum inhibitory concentration (MIC) values of linezolid were determined by broth microdilution and interpreted according to EUCAST guidelines (18), including the evaluation of MIC50 (minimum concentration that inhibits the growth of 50% of the isolates) and MIC90 (minimum concentration that inhibits the growth of 90% of the isolates). Interpretation of results for *E. lactis* and other non-*E*. *faecium* and *E. faecalis* isolates was done similarly as recent findings from EUCAST indicate that clinical breakpoints are valid across different enterococci species (20). *E. faecalis* ATCC 29212 was included as the control strain in all antimicrobial susceptibility tests. Isolates showing a resistance phenotype to three or more antibiotics belonging to different antibiotic families were considered multidrug-resistant (MDR) (21). Isolates from 2001 which are kept frozen in glycerol at −80°C were reanalyzed using current resistant breakpoints, and data comparison was done by sample.

### Genomic analysis and genetic context of linezolid resistance genes

A total of 44 isolates, including all categorized as MDR, as well as non-MDR representatives with variable antimicrobial susceptibility profiles from different species and samples, were selected for whole-genome sequencing (WGS): 20 *E. faecium,* 13 *E. lactis,* 10 *E. faecalis,* and 1 *E. thailandicus*. Genomic DNA was extracted using the Wizard Genomic DNA Purification kit (Promega Corporation, Madison, WI, USA) according to manufacturer’s instructions and its concentration evaluated by Qubit 3.0 Fluorometer (Invitrogen, Thermo Fisher Scientific, Waltham, MA, USA). WGS sequencing was performed by Illumina NovaSeq 6000 platform (2 × 150 bp) at the Eurofins Scientific (Germany). The raw and pre-processed data quality was checked using FastQC and the genomes were assembled with SPAdes (v.3.10.0). QUAST was used to evaluate the quality of genome assembly (22). Genes encoding antibiotic resistance, multilocus sequence typing (MLST), virulence, and plasmid content were screened using the most recent tools available at the Center for Genomic Epidemiology (CGE). A homemade database including 76 bacteriocin genes from *Bacillota* was also tested in these genomes (23). Clonal diversity was established through conventional MLST for a set of non-sequenced *E. lactis* (*n* = 11) and by cgMLST (Ridom SeqSphere+) in all sequenced isolates. Even though both typing methods are designed for *E. faecium* and not *E. lactis,* the *E. faecium* scheme was used to infer about *E. lactis* clonal diversity. We used a single nucleotide polymorphism (SNP)-based approach to compare our *E. faecium* and *E. lactis* genomes with all deposited in GenBank (available in June 2023) as *E. faecium* (*n* = 20.040) and *E. lactis* (*n* = 242) using the CSI Phylogeny tool from CGE. Isolates (*n* = 3) harboring *optr*A and/or *poxt*A genes were selected for long-read sequencing (Oxford Nanopore Technologies; Plasmidsaurus, USA). Hybrid assemblies of Illumina short reads and nanopore long-read data were generated with Unicycler (v.0.5.0), and mapping/annotation of *optr*A and *poxt*A contigs were performed using Geneious Prime (v.2.1). The *optr*A/*poxt*A-carrying platforms were mapped against similar ones (>90% nucleotide identity and coverage) found in strains collected from various sources and time periods using BLASTN and Easyfig (v.2.2.2) (24). Plasmid comparisons were achieved through BLAST Ring Image Generator (BRIG) (v.0.95) (25) and gene functions categorized based on the Clusters of Orthologous Groups of proteins (COGs) database of eggNOG (v.5.0) (26).

### Qualitative bacteriocin assay

All sequenced *E. faecium* (*n* = 20) and *E. lactis* (*n* = 13), along with selected clinical isolates from previous studies (*n* = 6), underwent a qualitative bacteriocin production and sensitivity testing (all-against-all) based on a soft-agar-overlay assay (27). The selected clinical isolates comprised four dominant vancomycin-resistant *E. faecium* (VREfm) clones (ST17, ST80, ST117, ST192), each with distinct bacteriocin profiles (carrying variable bacteriocin genes), and two *E. lactis* isolates (11). Briefly, 50 µL of the indicator overnight culture was added to 5 mL of molten soft tryptic soy broth at 45°C previously enhanced with 0.7% agar and 0.5% yeast extract and transferred onto a tryptic soy agar plate, supplemented with the same quantity of yeast extract. Afterward, with a sterile toothpick, a single colony of each isolate to be tested was transferred to the agar plate seeded with the indicator. Plates were incubated at 37°C for 48 h, and inhibition zones around the strains were measured at 24 h and 48 h (27).

### Statistical analysis

Statistical analysis was conducted in IBM SPSS Statistics 29, involving descriptive statistics and classic inferential statistics, namely χ^2^ and Fisher’s exact tests (28). *Enterococcus* species present in less than five positive samples were excluded. Predictor variables for binary logistic regression were chosen based on Fisher’s exact test results (*P* < 0.200; according to criteria used in previous studies [28], in order to avoid exclusion of potentially interesting variables in the model), excluding those with <5 positive observations, whereas age and sex variables were consistently included. Binary logistic regressions used the Wald backward method with thresholds of 0.05 for entry and 0.10 for removal, a classification cut-off of 0.5, and a maximum of 20 iterations. These criteria suited the exploratory nature of this work (i.e., identifying possible predictor variables for future analysis) and enhanced model accuracy compared to likelihood ratio. The study aimed to predict bacterial exposure risk based on predictors, with results shown as odds ratios (OR). Bootstrapping in R was employed as a resampling technique to determine the confidence interval for accuracy (28). Differences in antimicrobial resistance between 2001 and 2022 samples were analyzed by the χ^2^ test (α = 0.05).

## RESULTS

### Participant background and *Enterococcus* species colonization

Fifty-one fecal samples were collected from 29 women and 22 men, aged between 18 and 85 years old (mean age: 45 years; standard deviation: ±18.0). Most volunteers (98%) reported a diversified diet, with 18% consuming both raw or undercooked meat/fish and vegetables. Contact with pets (mainly cats and dogs) or farm animals was reported by 61% and 10% of the participants, respectively. Information regarding underlying diseases, supplementation, and traveling, among others is provided in Table S1.

Enterococci (*n* = 315) were recovered from all volunteers (1–13 isolates per sample). The species identified comprised *E. lactis* (75% of samples), *E. faecalis* (65%), *E. faecium* (47%), and *Enterococcus hirae* (20%). *E. durans*, *E. thailandicus*, *E. gallinarum,* and *E. casseliflavus* were each found only in a single sample (2% each) (Fig. S1). *E. lactis* was the most frequently detected species (*P* < 0.05), although no singles species was found in all samples. Participants were colonized by one (29%) to five enterococcal species (2%), with the most common colonization pattern involving two species (37%), predominantly *E. lactis + E. faecalis* (53% of the cases) (Figure S1).

In the 2001 collection, the predominant *Enterococcus* species was *E. faecalis* (66%), followed by *E. faecium* (59%) and *E. gallinarum* (3%). However, after further refining the differentiation between *E. faecium* and *E. lactis*, we found that of the 59% samples initially identified as harboring *E. faecium*, only 39% actually contained *E. faecium*, while 24% contained *E. lactis,* with some samples containing both species. In the current 2022 collection, focusing only on species retrieved from non-supplemented plates for non-biased comparison (51 samples; 87 isolates), we identified *E. faecalis* (63%), *E. lactis* (61%), and *E. faecium* (31%) species. The most significant difference between the two studies was the prevalence of *E. lactis,* which increased 2.5 times in the 20 years period, from 24% in 2001 to 61% in 2022 (*P* < 0.05).

Statistical analysis in the 2022 collection revealed that predictor variables, mostly related to dietary habits, were identified for *E. faecium, E. lactis,* and *E. faecalis* strains (Tables S2 and S3). Most predictor variables were protective (OR <1), except for “has diarrhoea without pain” (OR = 9.287) which was positively associated with *E. faecalis* presence. *E. faecium* was negatively correlated with “eats leafy vegetables” (OR = 0.333) whereas *E. faecalis* was negatively correlated with “eats raw fish” (OR = 0.117) and “eats root vegetables” (OR = 0.184) (Tables S2 and S3). The accuracy of the final models varied (64.0%–84.3%). The highest prediction accuracy (84.3%, with a range of 64.7%–94.1%) was found for the variables of being female (OR = 0.056), “eats white meat” (OR = 0.196), and “eats leafy vegetables” (OR = 0.187), all of which were negatively correlated with *E. lactis* (Table S3). Nonetheless, these results must be interpreted considering the sample size, the characteristics of the sampled population (predominantly middle-aged residents of the metropolitan area of Porto), and the qualitative nature of questions. To explore the potentially interesting relationship with white meat, we analyzed samples from 2001 (*n* = 59) that included data on the presence of *E. lactis*, *E. faecalis*, and *E. faecium*, along with records of white meat consumption. A negative relationship with *E. lactis* was also confirmed in the 2001 sample set (*P* = 0.030; Tables S4 and S5).

### Antibiotic resistance patterns in *Enterococcus* species: a comparative analysis of 2001 and 2022 data

**Fig 1:**
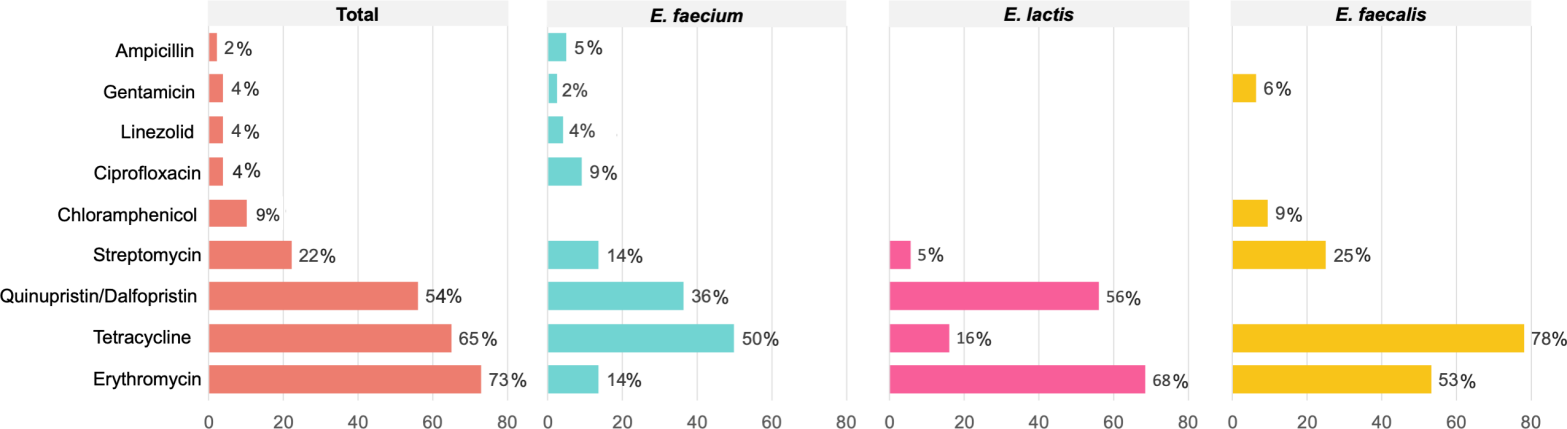
Percentage of antibiotic resistance by *E. faecium*, *E. lactis,* and *E. faecalis* species and cumulative across all tested antibiotics. Counts were made by sample (*n* = 51), and resistance to chloramphenicol was only evaluated in *E. faecalis* isolates.

**Fig 2:**
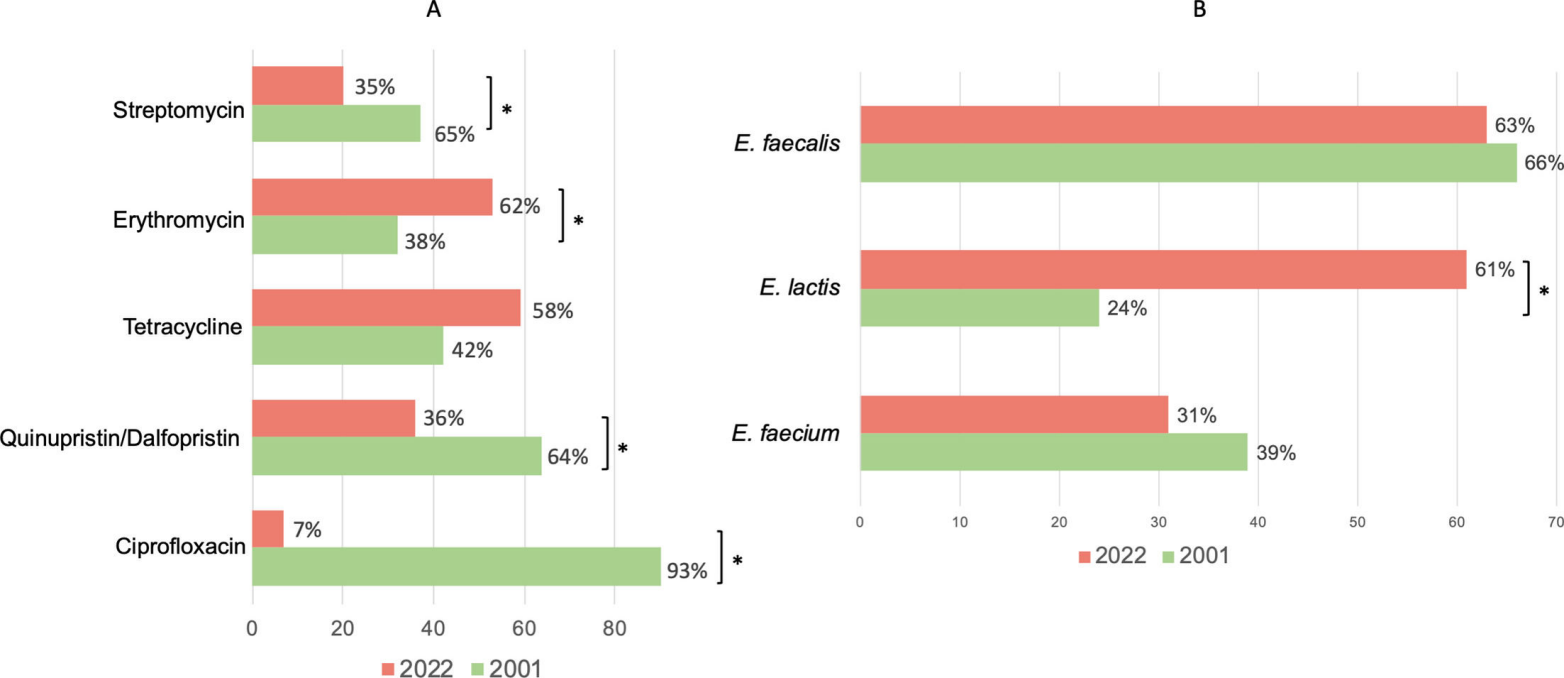
(**A**) Percentage of human fecal samples collected in 2001 (green bar) and 2022 (orange bar) that contain antibiotic-resistant *Enterococcus* spp. (**B**) Percentage of human fecal samples collected in 2001 (green bar) and 2022 (orange bar) that carry *E. faecalis, E. lactis,* and/or *E. faecium*. The percentages represent the proportion of samples carrying each of these species. Resistance to quinupristin-dalfopristin was only considered for *E. faecium* and *E. lactis* species. For all comparisons, only isolates recovered from non-supplemented Slanetz-Bartley plates were considered. *, *P* < 0.05 (Fisher’s exact test).

Most samples (92%) from 2022 contained isolates exhibiting resistant to a least one antibiotic, while only 8% of samples (*n* = 4) carried fully susceptible isolates of *E. faecalis, E. faecium,* and/or *E. lactis*. MDR isolates were identified in 24% of samples (12 in total), with *E. faecalis* being the predominant species (67% of MDR isolates). Antibiotic resistance rates varied among the different samples: erythromycin (73%), tetracycline (65%), quinupristin-dalfopristin (54%, only considering *E. faecium* and *E. lactis*), streptomycin (22%), chloramphenicol (13%; only tested in *E. faecalis* isolates), ciprofloxacin (4%), linezolid (MIC50 = 4 mg/L and MIC90 = 4 mg/L) and gentamicin (4% each), and ampicillin (2%) (Fig. 1). Significant differences in antibiotic resistance rates were observed between species (Fig. 1): *E. faecium* showed lower resistance to erythromycin compared to *E. lactis* and *E. faecalis* (14% vs 68% and 53%; *P* < 0.05). *E. faecalis* was more resistant to tetracycline than *E. lactis* (78% vs 16%; *P* < 0.05), while *E. lactis* was more resistant to quinupristin-dalfopristin than *E. faecium* (54% vs 36%; *P* < 0.05). When comparing antibiotic resistance rates between 2001 and 2022, an overall decrease is significant for ciprofloxacin (93% versus 7%; *P* < 0.05), quinupristin-dalfopristin (64% versus 36%; *P* < 0.05), and streptomycin (65% versus 35%; *P* < 0.05) (Fig. 2). In contrast, 2022 samples showed significantly higher resistance rates to erythromycin (38% versus 62%; *P* < 0.05) (Fig. 2). MDR rates were highly similar (29% in 2001 against 24% in 2022; *P* > 0.05). Linezolid, ampicillin and vancomycin resistance rates were not compared since isolates resistant to these antibiotics were only retrieved from antibiotic-supplemented plates in the current study.

The 2022 sequenced isolates (*n* = 44, representing 61% of samples) carried acquired genes putatively conferring resistance to aminoglycosides [*aph(3')-III*, *aac(6')-aph(2’’*), *ant(6)-Ia*, *str*, *aph(2'')-Ia*], macrolides-lincosamides-streptogramin B [*erm(B), lnu(B), lnu(G), lnu(A*)], lincosamides-pleuromutilins-streptogramin A [*lsa(E*)], tetracyclines [*tet(M*), *tet(L*)], vancomycin (*van*HAX), chloramphenicol (*cat*), oxazolidinones/phenicols [*optrA*6 (GenBank accession no. KT862784)*, poxtA*1 (GenBank accession no. MH746818)], phenicols (*fex*A*, fex*B), and trimethoprim (*dfrG*), and varying in their homology against reference strains (Table S6). *E. faecium* and *E. faecalis* carried between 0–11 and 0–10 acquired AMR genes, respectively, while *E. lactis* had the fewest, carrying only 0–3 genes [*str*, *tet(M*), and/or *tet(L*)], with most (10 out of 13) lacking any acquired genes present in the tested AMR database. All sequenced *E. faecium* (13–19 mutations), as well as five *E. lactis* isolates (1–5 mutations), were found to have mutations in the *pbp5* gene which have been associated with *pbp5* clade A1R and ampicillin resistance (Table S6). Although all isolates were predicted to exhibit a resistance phenotype by ResFinder 4.1, only one *E. faecium* isolate expressed resistance to ampicillin (MIC >256 mg/L). Only one *E. faecium* isolate carried mutations in the *gyrA* and *parC* genes. The single *E. faecium* harboring the *van*HAX cluster was susceptible to vancomycin (MIC = 4 mg/L), being thus considered a vancomycin-variable enterococci (VVE), and exhibited resistance to ampicillin, erythromycin, ciprofloxacin, gentamicin, and streptomycin. This isolate was obtained from an individual without hospital contact in the previous 12 months and corresponds to one of the two healthy volunteers answering as the most frequent consumed meat “only pork” and having frequent contact with different animals (dogs, rabbits, horses, goats, chickens).

### Linezolid-resistant isolates and genomic context of *optrA* and *poxtA* genes

**Fig 3:**
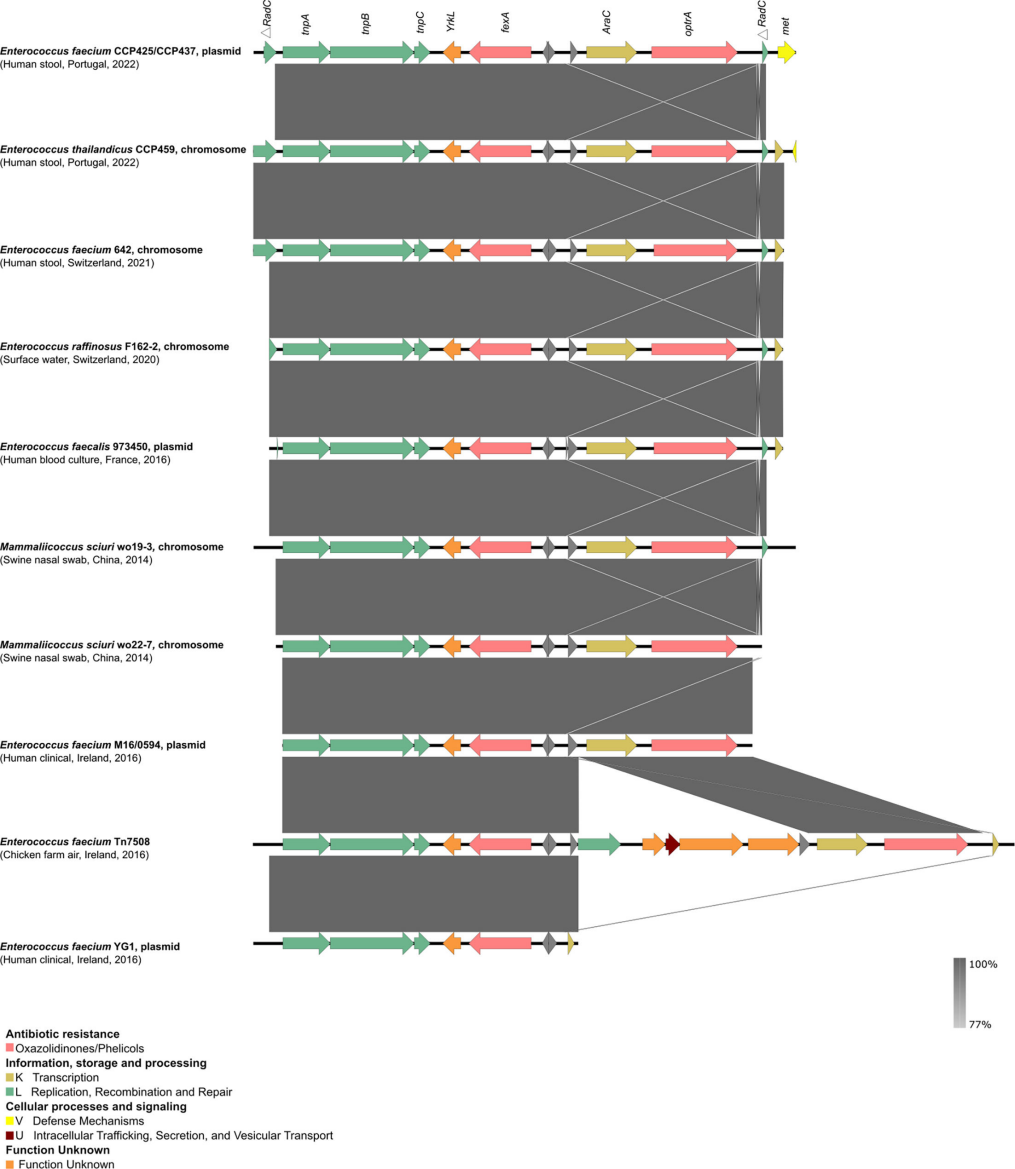
Schematic representation of *optr*A platforms in highly identical plasmids or chromosomal platforms present in the GenBank database. Colored arrows represent genes, and the direction of the arrows indicates their orientation. Gray arrows represent hypothetical proteins; the remaining colors represent the gene function according to the Clusters of Orthologous Groups database classification indicated in the key. The image was generated using the BLASTN option of Easyfig 2.2.2 (24) and based on eggNOG annotations (26).

All *optr*A- and/or *poxt*A-positive isolates exhibited clinical resistance to linezolid (MIC of 8–16 mg/L) (Table S6). These included two *E. faecium* isolates (ST128) from the same sample, both carrying *optr*A and *poxt*A on distinct plasmids. Additionally, these *E. faecium* isolates were resistant to chloramphenicol (*fex*A*, fex*B), and, in one case, also resistant to aminoglycosides [*ant (6)-la*, *aph(3')-III*], lincosamides-streptogramin B [*erm(B), lnu(B*)], and to lincosamides-pleuromutilins-streptogramin A [*lsa(E*)]. Furthermore, one *E. thailandicus* isolate from a different sample, which carried the *optr*A gene on the chromosome and lacked plasmids, was resistant to tetracycline [*tet(M*)] and chloramphenicol (*fex*A). The genetic context of the *optr*A gene in both *E. faecium* (plasmid-borne) and *E. thailandicus* (chromosomal) was highly identical (Fig. 3). Detailed data can be found in Table S6.

**Fig 4:**
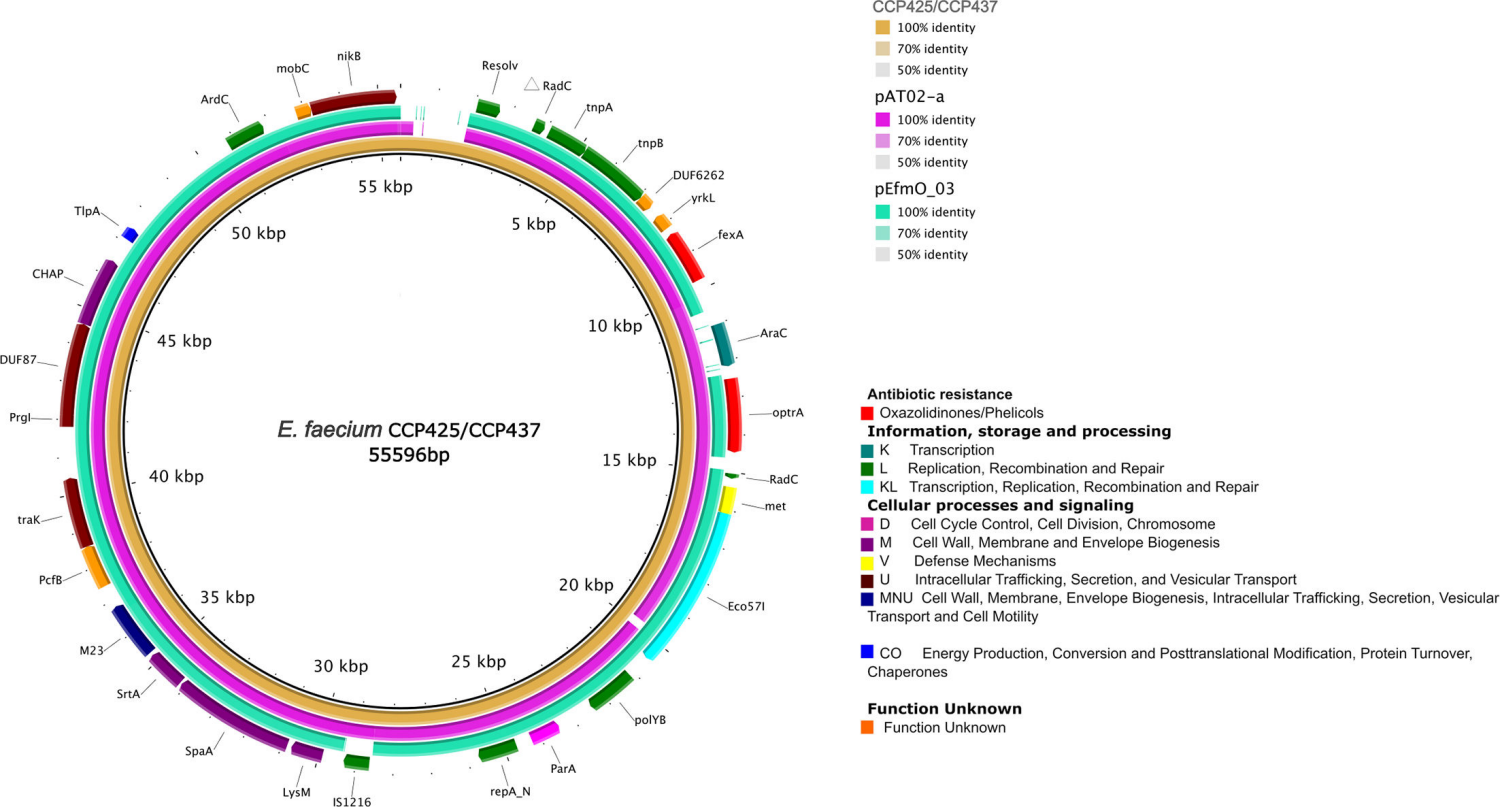
BRIG alignment of three *optr*A-carrying plasmids from different sources and countries. The novel *optr*A-carrying plasmid isolated from CCP425 and CCP437 isolates (deposited under GenBank accession no. CP161870 and CP161865, respectively) was annotated using Geneious Prime (v.2.1) and eggNOG-mapper (http://eggnog-mapper.embl.de) as reference plasmids. Each ring corresponds to a plasmid except the outermost ring which is an annotation of the reference plasmid and shows the direction of transcriptional open-reading frames. pAT02-a (GenBank accession no. CP097062.1) was found in a ST1846 *E. faecium* from pet food in Switzerland (2022) and pEfmO_03 (GenBank accession no. MT261365.1) was identified in a ST80 *E. faecium* recovered from a hospitalized patient in Ireland (2019). Genes encoding for antibiotic resistance (red), information, storage, and processing (teal-blue, green, and electric-blue), and cellular processes and signaling (pink, purple, yellow, brown, dark blue) are indicated.

The *optr*A genetic backgrounds in our *E. faecium* strains closely resembled those in *E. faecalis, E. raffinosus*, and *Mammaliicoccus sciuri* strains isolated from a range of sources (clinical, animal, human, and water) across multiple countries (China, Switzerland, France, and Ireland) from 2014 to 2021. Despite certain variations, such as the presence or absence of the DNA repair protein *rad*C, all *optr*A-containing platforms exhibited transposases (*tnp*A, *tnp*B, *tnp*C), an MFS transporter conferring resistance to phenicols (*fex*A), and the transcriptional regulator gene *ara*C. The Tn*7508*, part of the Tn*554* family, showed the highest degree of similarity to our platform structure, showing additional genes with unknown function upstream the *optr*A gene (Fig. 3). The plasmids carrying *optr*A and *fex*A of our *E. faecium* isolates were equal and sized 55.6 kbp (GenBank accession no. CP161870 and CP161865). They lacked other AMR genes and contained a replication protein belonging to the RepA_N family (repA) showing 100% nt identity to a few plasmids described in *E. faecium* strains obtained from hospitalized patients in China (pDY28 also carrying *optr*A; GenBank accession no. NZ_MW207670.1) and Ireland (pEFmO_03) or pet food in Switzerland (pAT02-a). The most similar plasmids (with 95%–91% coverage and 99% nt identity) were pAT02-a (GenBank accession no. CP097062.1) and pEfmO_03 (GenBank accession no. MT261365.1) which shared the same backbone besides plasmid replication genes. These plasmids were found to be carried by different *E. faecium* strains (ST1846 and ST80) obtained from pet food (Switzerland, 2022) and a hospitalized patient (Ireland, 2019), respectively (Fig. 4).

**Fig 5:**
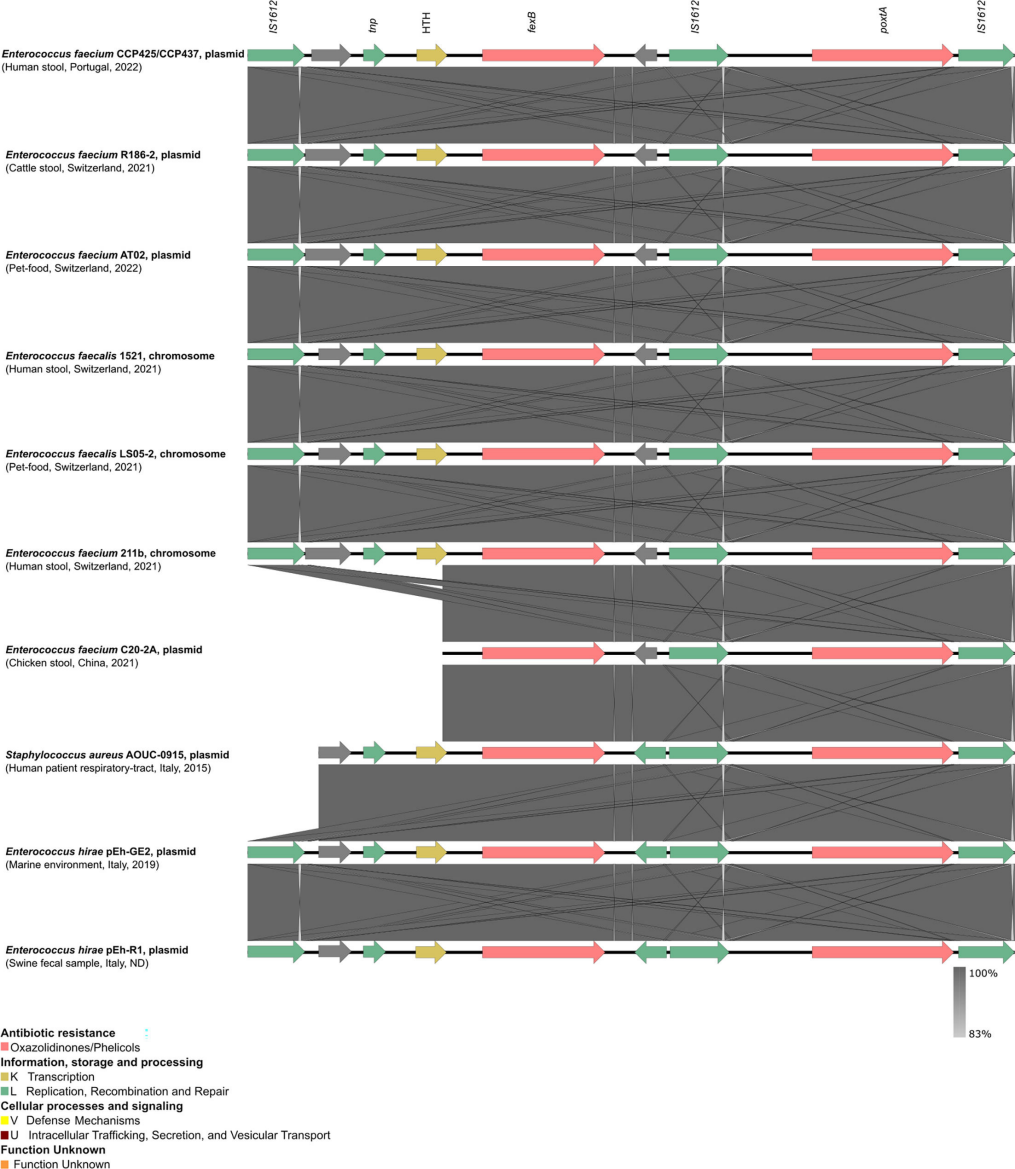
Schematic representation of *poxt*A platforms in highly identical plasmids or chromosomal platforms present in the GenBank database. Colored arrows represent genes, and the direction of the arrows indicates their orientation. Gray arrows represent hypothetical proteins of unknown function; the remaining colors represent the gene function according to the Clusters of Orthologous Groups database classification indicated in the key. The image was generated using the BLASTN option of Easyfig 2.2.2 (24) and based on eggNOG annotations (26).

**Fig 6:**
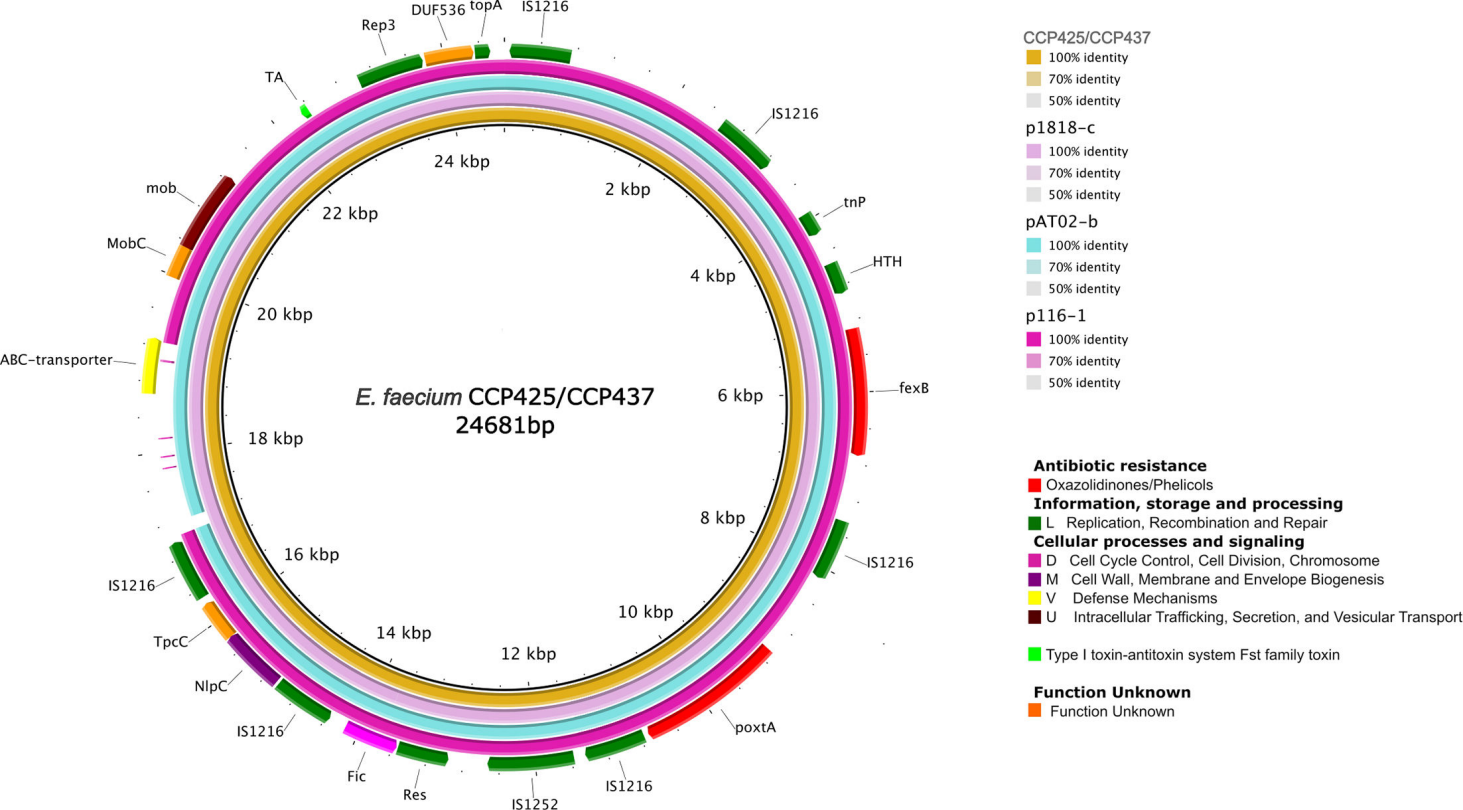
BRIG alignment of four *poxt*A-carrying plasmids from different sources and countries. The novel *poxt*A-carrying plasmid isolated from CCP425 and CCP437 isolates (deposited under GenBank accession no. CP161871 and CP161866, respectively) was annotated using Geneious Prime (v.2.1) and eggNOG-mapper (http://eggnog-mapper.embl.de) as reference plasmid. Each ring corresponds to a plasmid except the outermost ring which is an annotation of the reference plasmid and shows the direction of transcriptional open-reading frames. p1818-c (GenBank accession no. CP091209.1) was found in a ST29 *E. faecium* from human stool in Switzerland (2021), pAT02-b (GenBank accession no. CP097063.1) was identified in a ST1846 *E. faecium* recovered from pet food in Switzerland (2022), and p116-1 (GenBank accession no. CP047328.1) was isolated from a ST32 *E. faecium* recovered from a swine in China (2019). Genes encoding for antibiotic resistance (red), information, storage, and processing (dark green), and cellular processes and signaling (pink, purple, yellow, brown) are indicated.

The *poxt*A gene context showed high similarity with that of other *E. faecium, E. faecalis, E. hirae,* and *Staphylococcus aureus* strains*,* where it was either plasmid- or chromosomally located (Fig. 5). Most isolates were obtained from stool samples from both human and food-producing animals, but also from pet food, marine environment, and from a patient respiratory tract, collected in Switzerland, China, and Italy. The genetic contexts presented showed an overall conserved background as consistently demonstrated by the co-location of *poxt*A with *fex*B, a gene conferring resistance to phenicols (Fig. 5). The *poxt*A genetic background of our isolates was inserted on a 24.7-kbp plasmid (deposited under GenBank accession no. CP161871 and CP161866) containing a replication protein belonging to the Rep_3 family (rep29) (Fig. 6). We found three plasmids identical to ours (with 91%–100% coverage and 99% nt identity), p1818-c (GenBank accession no. CP091209.1), pAT02-b (GenBank accession no. CP097063.1), and p116-1 (GenBank accession no. CP047328.1). All plasmids were found in *E. faecium* strains with different STs isolated from human stool in Switzerland (2021), pet food in Switzerland (2022), and swine in China (2019), respectively. All plasmids shared the same backbone and plasmid replication genes and harbored several insertion sequences as IS*1216*-like (*n* = 7) and IS*1252*-like (*n* = 1) elements (Fig. 6).

### Phylogeny, virulome, and plasmidome of enterococci carriage isolates

A phylogenetic tree comprising the sequenced *E. faecium* and *E. lactis* genomes confirmed their clear separation with >24,714 SNPs apart (Fig. S2). Within *E. faecium* genomes, the ST78 VVE isolate clustered apart from the others as the single isolate belonging to clade A1. The sequence types of the 56 isolates, including the 44 sequenced enterococci plus 12 non-sequenced *E. lactis,* were established as a total of 45 STs: *n* = 18 (four novel) in 25 *E. lactis; n* = 18 (six novel) in 20 *E. faecium; n* = 9 in 10 *E. faecalis* (Tables S6 and S7). Several novel complex types (CTs) were identified in the three species. Some of the STs identified among our *E. faecium* and *E. lactis* isolates were previously related to human clinical (e.g., *E. faecium:* ST78, ST92, ST128, ST286; *E. lactis*: ST94, ST361), hospital surveillance/environment (e.g., *E. faecium:* ST32, ST78, ST165, ST292; *E. lactis*: ST94, ST107, ST361, ST717), community (*E. faecium:* ST32, ST47; *E. lactis*: ST60, ST94, ST361), environment (e.g., *E. faecium:* ST32; *E. lactis*: ST640), and animal or animal food (e.g., *E. faecium*: ST269; *E. lactis*: ST75, ST76) in different countries ranging from 1956 up to 2022 (pubmlst.org). Regarding *E. faecalis*, the ST16, ST21, and ST25 could be correlated with all the categories mentioned above. Furthermore, the cgMLST analysis, which compared our genomes with those from thousands available at the GenBank, only identified an overlap with our VVE isolate (ST78, CT230). Our genome showed 0–11 SNPs of difference compared to three other genomes related to the hospital setting (same ST and CT), including one from hospital surveillance in Portugal/2010 (GenBank accession number SAMN00779852) and two from human infections in Portugal/2009 and with the same VVE phenotype (Biosample accession no. SAMN44056311 and SAMN44056313).

Using the recently available virulence database specific for *E. faecium* and *E. lactis*, we observed a diverse array of putative virulence markers (PVMs) across the analyzed isolates. *E. faecium* isolates harbored between 8 and 23 PVMs, while *E. lactis* isolates contained 13–20 PVMs (Table S8). This analysis revealed both similarities and distinct patterns in the distribution of virulence factors between these closely related species. Overall, both *E. faecium* and *E. lactis* isolates were strongly associated with community gene variants rather than hospital ones, with exceptions in the *fms14* and *ebpC* genes. In several instances where hospital variants were present, the genes were actually truncated, as observed in *scm* and *ecbA*. Although *E. faecium* and *E. lactis* shared most PVMs and some gene variants, specific PVMs (*fms15*, *ptsD, orf1481*) were identified exclusively in *E. faecium* isolates. Certain gene variants (*scm, fnm, gls20, gls33, glsB, ccpA*) were strictly species-specific, with *E. lactis* gene variants present only in *E. lactis* and *E. faecium* gene variants (whether hospital- or community-associated) only in *E. faecium* isolates. Intriguingly, for many other genes, particularly *acm, sagA,* and *fms20*, *E. lactis* gene variants were found to intermix with both community and hospital variants of *E. faecium* in both species.

The ST78 *E. faecium* isolate was the most enriched in PVMs, primarily corresponding to hospital gene variants, including markers of the *E. faecium* hospital-associated clade A1 (*ptsD, orf1481, sgrA,* and hospital variants of the *fms14-fms17-fms13* gene cluster) (Table S8).

Using the same database, we observed that the single *E. thailandicus* isolate harbored only a few genes, but they were similar to those found in *E. faecium,* including the hospital marker *ptsD*.

In contrast, *E. faecalis* isolates displayed a different but generally consistent virulence gene profile across the sequenced isolates. Most of the genes found were of biofilm formation (*srtA, ebpA, ebpC*), adhesins (*ace*), surface protein (*efaA*), gelatinase (*gel*), among others (*ElrA*, *cCF10, cOB1, cad, came, tpx*), but only a few presented 100% identity with reference strains included in the database. Notably, four *E. faecalis* isolates, including those belonging to ST16 and the related ST179, presented genes coding for cytolysin subunits and secretion functions (*cylA, cylL, cylM*) (Table S8).

The plasmidome analysis of sequenced isolates revealed six plasmid families: RepA_N, Inc18, Rep1, Rep2, Rep3, and Rep_trans, comprising 28 rep types in total (Table S9). The Inc18 family was the most diverse, with nine rep types, while Rep2 was represented by a single type exclusive to *E. faecium*. Several rep types were shared between species: repA(pNB2354p1) [RepA_N], repE(pAMbeta), repE(pKL0018), repE(pIP816) [Inc18]; repB(pUB110) [Rep1]; and repA(p200B) [Rep3] were common to *E. faecium* and *E. lactis*, while rep2(pRE25) [Inc18] was shared among all three species. The number of rep types varied by species, with *E. faecium* and *E. lactis* carrying zero to six types each, and *E. faecalis* zero to four types. Notably, 12 of the 28 rep types were found only in the truncated form. Two rep genes of clinical significance were identified: repA(AUS0004p1) in the ST78 *E. faecium* isolate and repA(pMG2200) in the ST179 *E. faecalis* isolates, both previously associated with plasmids harboring linezolid and vancomycin resistance genes in human infections.

### Bacteriocin gene repertoire and antimicrobial inhibition assays reveal a complex interplay between *E. faecium* and *E. lactis*

Our analysis revealed a diverse bacteriocin gene repertoire across the *Enterococcus* species studied, with a total of 22 different bacteriocin genes identified, including seven novel ones. The distribution of these genes varied significantly among species, with *E. lactis* showing the highest prevalence (92% of isolates), followed by *E. faecium* (76%) and *E. faecalis* (22%).

**Fig 7:**
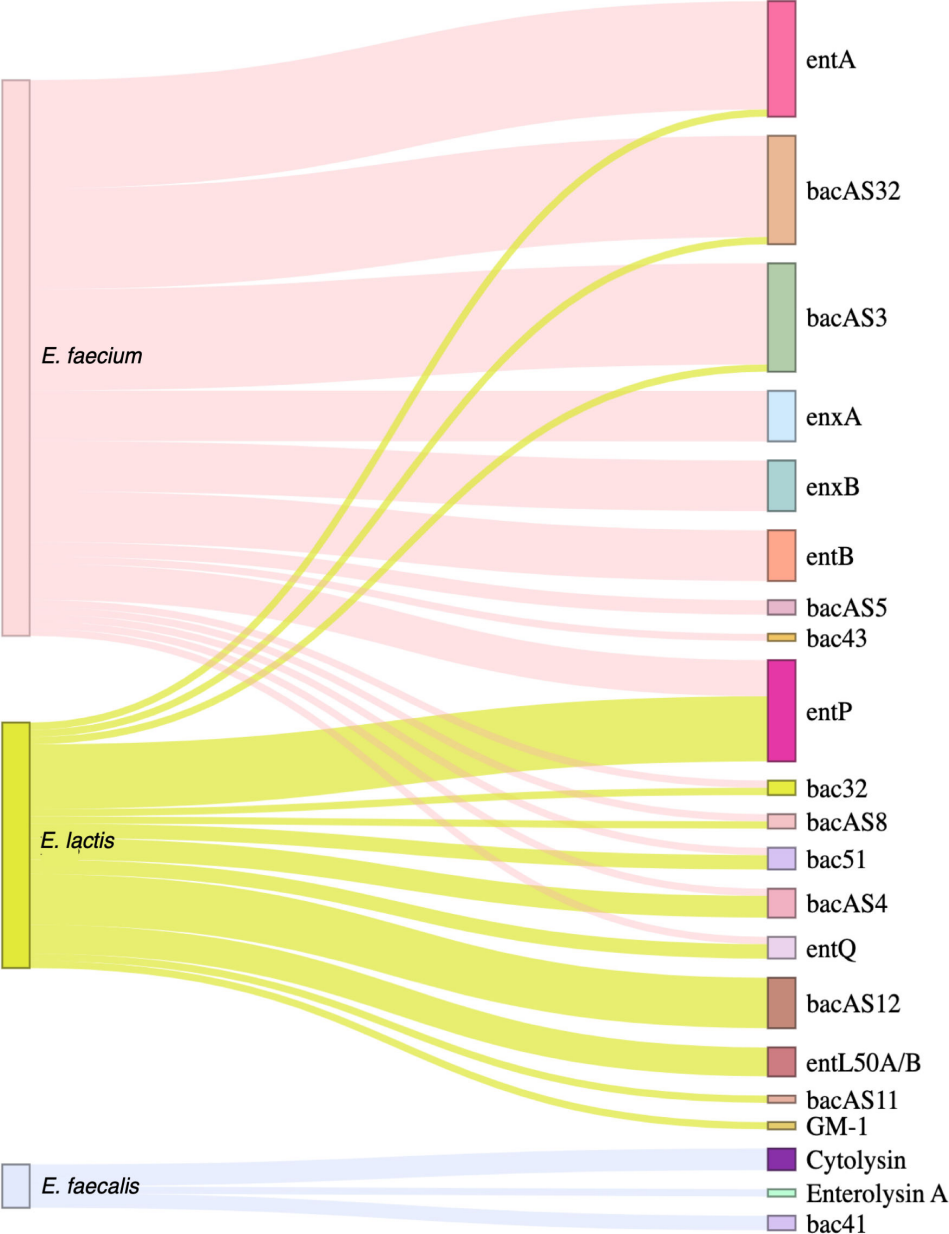
Sankey diagram representing, from right to left, the occurrence and diversity of bacteriocin genes by *Enterococcus* species, using the *Bacillota* bacteriocin database (23). The width of each connection is proportional to the number of positive hits. The Sankey diagram was generated in R using the package networkD3 v.0.4 (29).

*E. faecium* isolates demonstrated the greatest diversity in bacteriocin genes, harboring up to nine bacteriocins per isolate (average of four per isolate), while *E. lactis* up to five (average of three), and *E. faecalis* with a maximum of two bacteriocin genes per isolate (average of 0.2) (Fig. 7; Fig. S3).

We observed species-specific bacteriocin genes, with *E. faecium* possessing five exclusive ones (*ent*B, *enx*A, *enx*B, *bac*AS5, and *bac*43), *E. lactis* four (*bac*AS12, *ent*L50A, *ent*L50B, and GM-1), and *E. faecalis* two (cytolysin and *bac*41). Notably, nine bacteriocin genes were shared between *E. faecium* and *E. lactis* (Fig. 7; Fig. S3). The diversity of bacteriocin genes content was highlighted by the different profiles displayed, with *E. lactis* and *E. faecium* exhibiting 11 different bacteriocin profiles, while *E. faecalis* only showed two (Fig. S3). The most prevalent bacteriocins were enterocin A in *E. faecium* (75% of isolates), enterocin P in *E. lactis* (69%), and cytolysin in *E. faecalis* (30%). (Fig. 7; Fig. S3). We observed consistent co-occurrence patterns of certain bacteriocin genes, often located on the same contig. For example, in *E. faecium* isolates, the presence of the *enx*A gene was invariably associated with *enx*B and *ent*B. Similarly, *bac*AS32 frequently co-occurred with *bac*AS3 in both *E. faecium* and *E. lactis*, while *ent*L50A was frequently paired with *ent*L50B in *E. lactis*.

**Fig 8:**
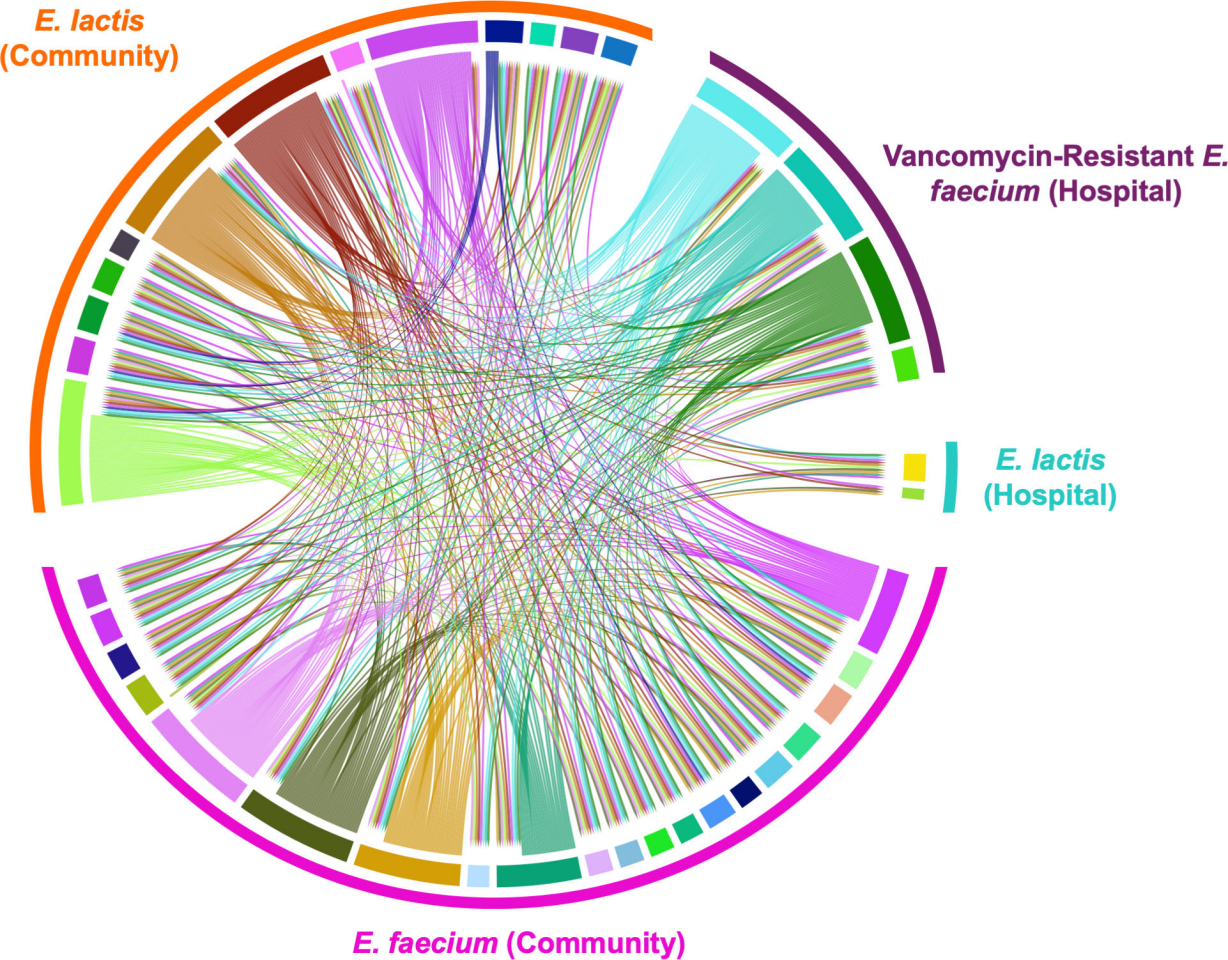
Chord diagram representing the inhibitory activity of the isolates tested. Each rectangle with a different color represents a different isolate. The isolates are grouped according to species (*E. faecium* or *E. lactis*) and its source (hospital or volunteers’ stool). The size of each rectangle is proportional to the number of isolates it can inhibit and/or the number of isolates that are inhibiting it. Arrows indicate the direction of inhibition.

Antimicrobial inhibition revealed that approximately 40% of isolates, including both *E. faecium* and *E. lactis*, exhibited inhibitory activity against at least one other strain. Conversely, all isolates were susceptible to inhibition by at least one other isolate (Fig. 8; Table S10). Interestingly, in four out of six cases where *E. faecium* and *E. lactis* co-colonized the same individual, these species did not inhibit each other, while in two cases, *E. lactis* inhibited *E. faecium* unilaterally.

The presence of bacteriocin genes did not always correlate with inhibitory activity. While all but one isolate lacking known bacteriocins failed to inhibit others, 42% of isolates carrying one to eight bacteriocin genes also showed no inhibitory activity. *E. lactis* demonstrated a slightly higher inhibitory potential, with 46% of strains inhibiting at least one other isolate, compared to 35% of *E. faecium* strains. Notably, 25% of commensal isolates (21% *E. faecium* and 31% *E. lactis*) with diverse bacteriocin profiles and sequence types showed inhibitory activity against at least one VRE isolate. Conversely, three out of four tested VRE isolates demonstrated broad inhibitory activity, affecting 93% of *E. faecium* and 74% of *E. lactis* commensal isolates (Fig. 8; Table S10). The VRE isolate without inhibitory activity possessed only the *ent*A bacteriocin gene. Moreover, a single *E. lactis* fecal isolate (ST361) was able to inhibit all strains tested including VRE and *E. lactis* from the clinical setting, and only two isolates (one *E. faecium* and one *E. lactis*) were able to inhibit this bacteriocinogenic strain carrying five different bacteriocin genes (*bac*AS12*, ent*P*, ent*L50A/B*, bac*AS8). Another clinical *E. lactis* with the same ST but without bacteriocin genes did not inhibit any of the tested isolates. Overall, isolates with more bacteriocin genes were less commonly inhibited, and similar bacteriocin profiles showed similar inhibition patterns (Fig. 8; Table S10). No correlation was found between the type of inhibitory spectrum and clonality.

## DISCUSSION

This study provides valuable insights into the *Enterococcus* fecal carriage in healthy humans, revealing that the community remains a significant reservoir of antibiotic-resistant enterococci harboring a complex mix of clinically relevant AMR, virulence, and bacteriocin genes. To the best of our knowledge, this is the first study to accurately identify the occurrence of *E. lactis* alongside *E. faecalis, E. faecium*, and other enterococci species in healthy volunteers. This precise identification demonstrates that *E. lactis* appears in a significant majority of samples, which would otherwise be underreported.

While it has been recognized that the former *E. faecium* clade B (now *E. lactis*) is present in the human gut, its true prevalence and significance have been underappreciated due to identification challenges. Most available studies have not distinguished between *E. faecium* and *E. lactis*, as this distinction has only recently become possible through phylogenomic analysis. Consequently, with accurate identification, existing reports are likely to undergo significant revisions. This was exemplified in our group’s previous study, where subsequent distinction between *E. faecium* and *E. lactis* revealed that a substantial portion of initially identified *E. faecium* isolates were, in fact, *E. lactis* (2). Among the few studies that have differentiated between enterococci species (4, 8, 30), results have varied, likely due to differences in methodological approaches, including sample pre-enrichment techniques and the use or absence of antibiotic selection. However, our findings, along with recent phylogenomic studies, suggest that *E. lactis* may be more prevalent in the human gut than previously thought. This emerging picture challenges earlier assumptions about *E. faecium*’s dominance and highlights the need for more comprehensive, species-specific investigations to accurately determine the relative abundance and distribution of these *Enterococcus* species in the human gut microbiome.

The rise in *E. lactis* prevalence from 24% in our 2001 surveillance study to 61% in the current study is noteworthy and warrants further investigation. However, we cannot discard a potential bias in the different sampling method (swab vs stool) between the two studies. Different studies have examined the differences between stool collection and swabs in terms of microbiological content and diversity, with most concluding that there are no significant discrepancies (30, 31). Given that *Enterococcus* species predominantly inhabit the ileum and colon, it is still plausible that stool collection captures a higher diversity of *Enterococcus* (32). Even if methodological differences could explain variations in recovery rates, our study still highlights the importance of accurate species identification in enterococci research. A significant knowledge gap persists regarding the influence of diet on *Enterococcus* spp. colonization in humans, particularly concerning the co-colonization of different enterococci species and the composition of antibiotic resistance genes. A recent study of 290 healthy adults in the USA suggested that individuals consuming diverse diets high in fiber and low in animal protein harbored fewer antibiotic resistance genes (33). The negative correlation between *E. lactis* and chicken meat consumption in both 2001 and 2022 studies raises intriguing questions about dietary influences on enterococci colonization. However, several limitations constrain our understanding. The prevalence of different enterococci species in various types of meat remains poorly characterized. Furthermore, our dietary assessment lacked the depth necessary to thoroughly evaluate the volunteers’ food consumption patterns in terms of both frequency and quantity. Additional factors that may influence results include age, sex, lifestyle, environmental exposures, medication use, health conditions, and sampling methodology. These limitations underscore the need for more comprehensive studies that consider a broader range of factors and employ more detailed dietary assessments. Such research would help elucidate the complex relationships between diet, enterococci colonization, and antibiotic resistance gene carriage in the human gut microbiome.

Our findings on MDR enterococci prevalence (24%) mirror those from 2 decades ago (29%). However, comparing these data with the limited number of similar studies is challenging due to variations in methodological approaches, differing definitions of MDR, and/or calculations of MDR per isolate rather than per sample (34, 35). The finding of linezolid resistance genes in different enterococci species suggests an environmental/food chain role in this acquisition as these genes have not been previously identified in clinical enterococci in our region. Notably, this study represents one of the few reports of the *optr*A gene in healthy volunteers (and the first in Portugal). ST128 *E. faecium* also carrying *optr*A *+ poxt*A has been described in the air of a swine farm in Spain (36). In this and other studies, enterococci carrying clinically relevant genes have been linked to regular contact with animals, raw meat, and/or untreated water, suggesting food chain as a possible source for this transmission (13, 37). Curiously, contact with animals was not identified as a statistically significant risk factor for the carriage of MDR isolates in our study. The presence of enterococci harboring *optr*A*/poxt*A on genetic platforms found in both clinical isolates and livestock across various countries, without an apparent relationship, strongly supports the mobile nature of these genes and suggests co-transmission events across different species, hosts, and regions. The first report of linezolid resistance in healthy humans in Portugal was reported in 2020, involving a 2001 *E. faecium* strain carrying *poxt*A (38). Although not frequent, the presence of these genes in *E. faecium* colonizing healthy humans is not unheard of as it was also described in the community in Switzerland (14) and China (39). Current linezolid resistance rates in hospital infections remain generally low (40), but different European countries have been reporting a gradual increase in linezolid-resistant enterococcal infections (41, 42). Additionally, linezolid consumption is increasing globally (43, 44) and the linezolid drug market is expected to grow significantly in the coming years (45). In parallel, an increase in amphenicol sales has been noted from 2011 to 2020 in European countries, including Portugal (46). The number of enterococci carrying acquired linezolid resistance genes among different hosts and countries is indeed increasing, including in food-producing animals of our region (38, 47–49). While the directionality of AMR transfer between humans and animals remains a subject of debate (50), evidence, including findings from our study, suggests that the food chain contributes to the reservoir of AMR genes in the human gut. This does not diminish the urgent need for judicious antibiotic use in humans. Recently, significant measures have been implemented in the EU, such as restricting the use of linezolid in humans and limiting off-label use of several antibiotics, including phenicols, in animals (51). In this sense, targeted and holistic One Health-based measures to preserve the efficacy of critical antibiotics for human medicine as linezolid are urgently needed to curb co-selection of linezolid resistance genes by the use of phenicols in livestock and oxazolidinones in human medicine.

The identification of a vancomycin-variable *E. faecium* isolate in a healthy human is particularly significant, as existing reports primarily involve hospital isolates (52–54). This finding, along with the emergence of VVE strains in various locations (53–56), underscores the need for screening *van* genes in every patient infected with an *E. faecium* strain to prevent underdetection and silent outbreaks. Finally, the statistically significant increase in erythromycin resistance from 2001 to 2022 likely reflects macrolides extensive use in the community and animal production (57). The frequent occurrence of genes conferring resistance to erythromycin and other macrolides within variable genetic clusters, along with various resistance genes (e.g., tetracyclines, aminoglycosides, oxazolidinones), may facilitate the co-selection and the continuous maintenance of such genetic platforms, especially under diverse antibiotic selective pressures.

*Enterococcus* spp. are well known as one of the greatest bacteriocin producers, both in number and diversity, a fact greatly supported by our results. The species specificity of certain bacteriocin genes aligns with a previous report by Tedim et al. in a collection of international deposited genomes and suggests potential roles in niche adaptation and competition (23). Particular bacteriocin genes (*bac* genes) have been previously described in significant hospital VRE clones such as ST117, ST17, and ST80 *E. faecium* (*bac*51, *bac*32 and *bac*43; [58–60]) and *E. faecalis* (*bac*41; [61]), or were also found in healthy students in Japan (*bac*43, *bac*32; [58, 59]). The diversity of bacteriocin genes and their correlation with inhibitory activity against other strains, in particular between *E. faecium* and *E. lactis,* also highlighted that a higher number of bacteriocin genes tended to be associated with stronger inhibition profiles (more strains inhibited). The finding that isolates with more bacteriocin genes were generally less susceptible to inhibition further suggests a potential role for these genes in microbial competition and adaptation. While the inhibitory capacity of *E. faecium* and *E. lactis* is evident and both species harbor numerous bacteriocin genes, assessing whether this inhibition was attributed to bacteriocins was beyond the scope of this study. Additionally, even though we tried to correlate bacteriocin genes with inhibition patterns, we did not delve into understanding if and how these genes were regulated or expressed.

The colonization dynamics between enterococcal commensals and healthcare-related isolates in the GI tract has been sporadically studied (62). *In vitro* studies showed that *E. faecium* clinical isolates, particularly VRE, have a higher inhibitory activity than human fecal isolates (27, 63). Our findings align with that from Wagner et al. describing that 76% of hospital isolates inhibited the commensal ones, whereas only 36% of the commensal isolates were able to inhibit the clinical ones (63). The dual existence of enterococci as a commensal and a clinical pathogen poses challenges in such analysis, but *in vivo* studies demonstrated that resistant isolates outcompete sensitive enterococci in the context of antibiotic pressure, but in its absence, isolates from the previous clade B outcompete those from subclade A1 (15, 64). Our results further suggest the complex interactions within the microbial community and the potential role of bacteriocins in microbial competition and adaptation (62). Numerous studies have demonstrated that specific purified bacteriocins can inhibit the growth of VRE (65, 66), a finding that is supported by our individual all-against-all assays. While these assays do not fully capture the intricate dynamics of the gut microbial network, they do shed light on the potential of both commensal *E. faecium* and *E. lactis* to inhibit clinical VRE, which may contribute to microbiota restoration after antibiotic treatments, and *vice versa*. This likely occurs under a delicate balance influenced by various factors, namely influencing bacterial fitness cost (67, 68).

To conclude, this study uncovers significant insights into the co-occurrence of the prevalent *E. lactis, E. faecalis,* and *E. faecium* species in the human gut. We acknowledge our sample size is limited; however, our results clearly highlight the importance of continuous surveillance of the human gut microbiota, given that these strains act as a significant source for the continuous A1 adaptation (69). Existing literature lacks comprehensive studies on intestinal colonization by antibiotic-resistant bacteria, with recent studies being geographically limited (70). Despite the surge in gut microbiome research, this usually reaches low taxonomic resolution (maximum species), with data on strains and their genomic content remaining scarce. Focus should also shift toward the community’s linezolid-resistant reservoir, rather than disproportionately targeting VRE, to preserve linezolid efficacy. Current and future trends could be predicted, for instance, through urban sewage metagenomics (71, 72) that target AMR genes and others of medical and public health significance. Such targeted surveillance can be complemented with innovative interventions involving diet or physiological biomolecules to increase bacterial stress or fitness cost as recently proposed (67). Given the known pre-colonization of the gastrointestinal tract by clinically significant enterococci, it is crucial to assess the community burden of AMR for effective surveillance and One Health action plans.

## SUPPLEMENTAL MATERIAL

Supplemental figures aem.01699-24-s0001.pdf Figures S1 to S3.

Supplemental text aem.01699-24-s0002.pdf Questionnaire used in this study.

Supplemental tables aem.01699-24-s0003.xlsx Tables S1 to S10.

ASM does not own the copyrights to Supplemental Material that may be linked to, or accessed through, an article. The authors have granted ASM a non-exclusive, world-wide license to publish the Supplemental Material files. Please contact the corresponding author directly for reuse.
