# Supplementary material for: The healthy human gut can take it all: vancomycin-variable, linezolid-resistant strains and specific bacteriocin-species interplay in Enterococcus spp
Source: Appl Environ Microbiol. 2024 Dec 19;91(1):e01699-24. doi: 10.1128/aem.01699-24 (PMC11784074; doi:10.1128/aem.01699-24)
Supplement: Supplemental figures — Figures S1 to S3. [file aem.01699-24-s0001.pdf]

SAMPLE	*E.lactis*	*E.faecium*	*E. faecalis*	*E.hirae*	*E. gallinarum*	*E. casseliflavus*	*E. durans*	*E. thailandicus*
1	54%	38%	8%					
2	57%	43%						
3			100%					
4	75%	25%						
5	50%	42%		8%				
6	100%							
7		100%						
8	100%							
9	67%		33%					
10	17%	17%	67%					
11	44%		44%	12%				
12	80%		20%					
13			75%	25%				
14	57%	43%						
15	25%		75%					
16	100%							
17	22%	33%	45%					
18	89%		11%					
19	100%							
20	50%		38%			13%		
21	75%	25%						
22	80%		20%					
23	60%	40%						
24	11%	11%	78%					
25	33%		67%					
26		60%	40%					
27	29%	14%	57%					
28			100%					
29	18%	18%	27%	27%	9%			
30		100%						
31	100%							
32	0%		100%					
33	72%	14%	14%					
34	70%	10%	20%					
35		100%						
36	100%							
37	50%		50%					
38	17%		83%					
39	33%		67%					
40	50%	13%	25%	13%				
41	100%							
42	50%		25%	13%				13%
43		40%	20%	20%			20%	
44			100%					
45	67%	11%	22%					
46	20%	20%	60%					
47	25%			75%				
48		57%		43%				
49	20%		60%	20%				
50			100%					
51	25%		75%					

Figure S1. Percentage of enterococcal species distribution per sample.

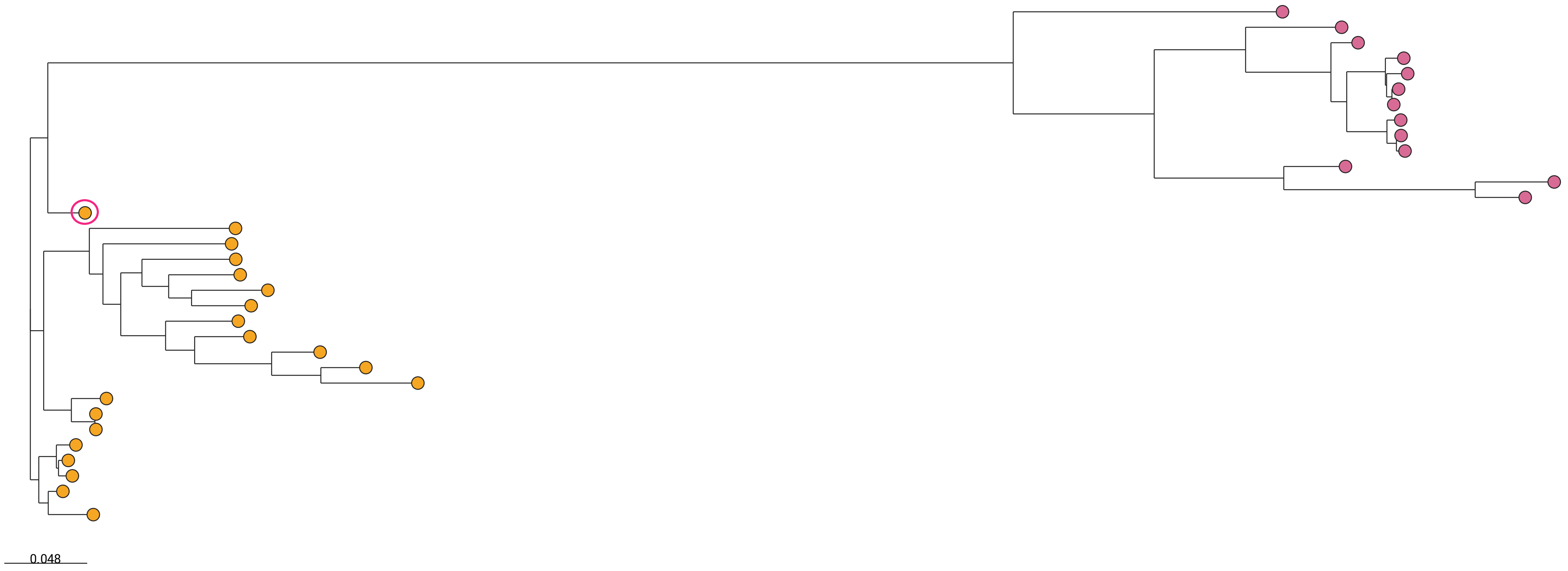

Figure S2. Whole-genome SNP-based phylogenetic tree of *E. faecium* and *E. lactis* isolates. Each circle represents a different isolate, and different colours indicate the corresponding species. The vancomycin-variable *Enterococcus faecium* (VVE) isolate is highlighted by a pink outer-circle. Genomes were mapped against *E. faecium* DO reference strain (GenBank accession assembly no. GCA_000174395.2) to infer a phylogeny based on the concatenated alignment of high-quality single nucleotide polymorphisms (SNPs) using CSI Phylogeny 1.4 of the Center for Genomic Epidemiology (https://cge.cbs.dtu.dk/services/CSIPhylogeny). Input parameters were: 10 × minimum depth and minimum relative depth at SNP positions; SNPs were filtered out if they were called within the vicinity of 300 bp of another SNP (pruning); minimum SNP quality of 30; minimum read mapping quality of 25; and minimum Z-score of 1.96. The tree illustration was created using Microreact (https://microreact.org/).

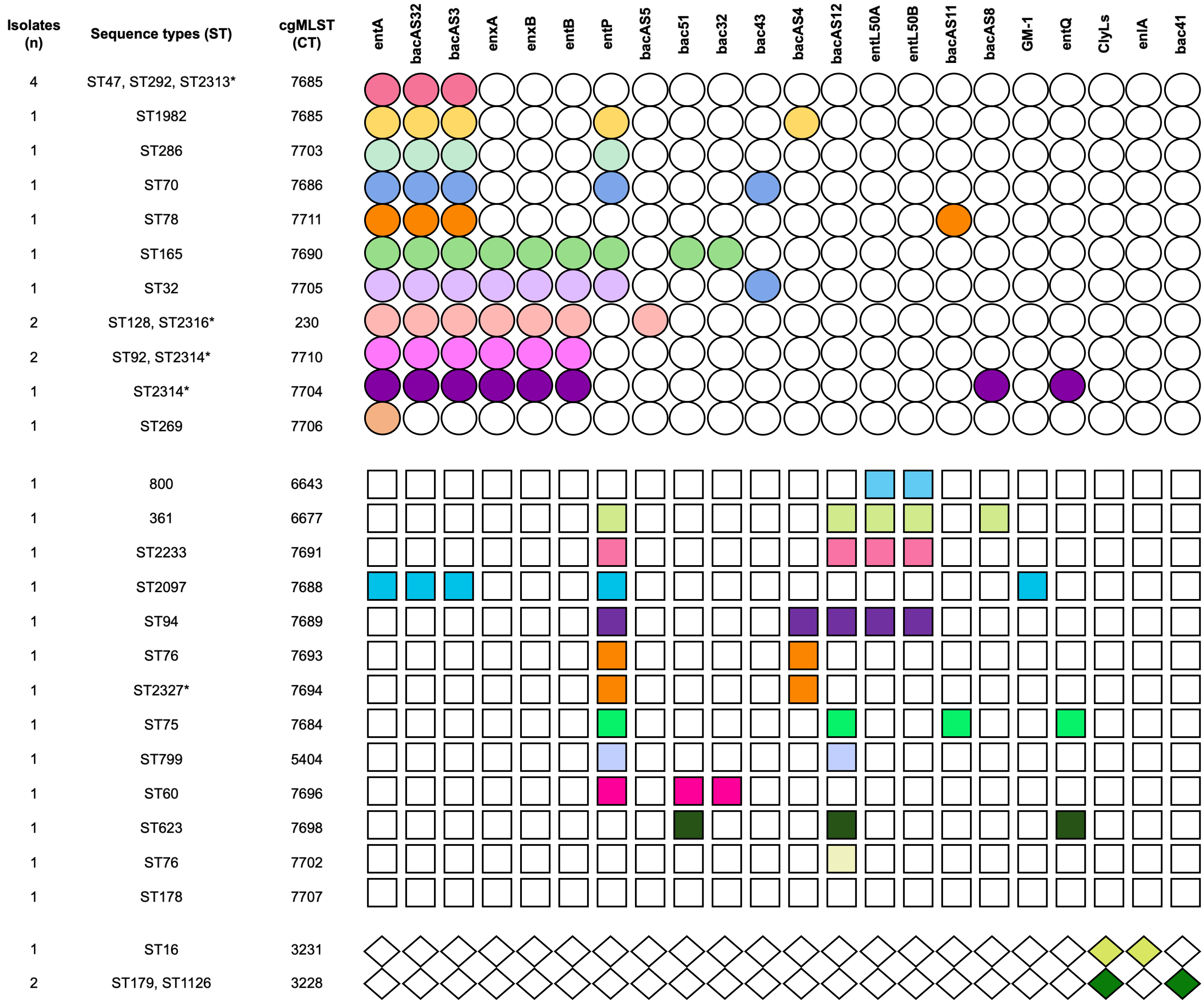

Figure S3. Profiles of bacteriocins genes per isolate from each species. Isolates with the same bacteriocin profile are grouped. Coloured cells represent the presence of the correspondent bacteriocin gene. Bacteriocin genes are coloured by bacteriocin profile. Circles correspond to *E. faecium*, squares to *E. lactis* and diamonds to *E. faecalis*.

*, means novel ST.
