# Supplementary material for: The healthy human gut can take it all: vancomycin-variable, linezolid-resistant strains and specific bacteriocin-species interplay in Enterococcus spp
Source: Appl Environ Microbiol. 2024 Dec 19;91(1):e01699-24. doi: 10.1128/aem.01699-24 (PMC11784074; doi:10.1128/aem.01699-24)
Supplement: Supplemental text — Questionnaire used in this study. [file aem.01699-24-s0002.pdf]

Information for Participants

Faecal carriage of multidrug-resistant bacteria by Portuguese healthy humans

Principal investigator: Prof. Ana R. Freitas Contact (e-mail): anarpf@gmail.com

◊ The main objective of this study is to characterize antibiotic-resistant bacteria in healthy individuals;

◊ Participation in this study involves completing a questionnaire (Annex 1) which will be done online whenever possible (depends on the volunteer);

◊ This participation does not present any risks to the Participant's Health, as non-invasive collection methodologies will be used;

◊ This study will contribute to a better understanding of the diversity of multidrug-resistant bacteria associated with the intestinal tract;

◊ Participation in the study is voluntary;

◊ The participant has time to reflect on the request for participation, including being able to hear opinions from family and/or friends;

◊ The participant has complete freedom to decide (yes or no) about their participation in the study;

◊ It is possible at any time to refuse or abandon participation in the study;

◊ Anonymity and confidentiality of the data obtained are guaranteed;

◊ This study was approved by the Ethics Committee of the Faculty of Pharmacy, University of Porto.

Disclaimer: This questionnaire has been translated into English for the reader's better understanding. When administered to participants, it was presented in their native language (Portuguese).

ANNEX 1

Date___________________ **Sample number**_________________________

Date of birth____________________(dd/mm/yy)

Sex: Female ☐ Check if you are in menopause ☐

Male ☐

Weight:___________(Kg) **Height:**___________(cm)

E-mail ____

City of residence________________________________ **Nationality** ____________________

Profession ____

Recent trips outside the country (last three months) No ☐ Yes ☐ Country __________

In the last year, did you experience any of the following symptoms?

Diarrhoea with abdominal pain ☐

Diarrhoea without abdominal pain ☐

Vomiting ☐

General malaise associated with vomiting and/or diarrhoea ☐

I did not experience any of the before mentioned symptoms ☐

	Poor	Fair	Good	Very good	Excellent
**In general, how do you consider your health?**	☐	☐	☐	☐	☐

Indicate if you have any of the following conditions:

☐	Diabetes	☐	Hypertension
☐	Heart disease	☐	Autoimmune disease
☐	Kidney disease	☐	Hyperthyroidism
☐	Liver disease	☐	Hypothyroidism
☐	Lung disease	☐	Chronic intestinal disorders
☐	Oral cavity disease	☐	Other(s) ________

Have you taken any antibiotic(s) in the last three months?

Yes ☐ Which one?________________ Date of last dose:______________________

No ☐

Regular chronic medication (example: antihypertensive, antidepressant, contraceptive pill, antacids, etc.):

Yes ☐ Which one(s)?____

____

No ☐

Do you take dietary supplements (e.g., vitamins, calcium, magnesium, etc.):

Yes ☐ Which one(s)?____

____

No ☐

Do you have any chronic disease that makes you go to the hospital or other health unit frequently?

Yes ☐ Which one?______________________How many times per year?_________

No ☐

Were you hospitalized in the last 12 months?

Yes ☐ How long?________________________Where?____________________

No ☐

Do you usually consume/contact untreated water:

Daily Weekly ☐

Monthly ☐

Sporadically ☐

Never ☐

Do you consume undercooked or raw meat or fish?

Yes ☐ Which ones?____

No ☐

What type of meat/charcuterie do you consume most frequently? ______________________

Do you frequently consume raw vegetables?

Yes ☐ Which ones?____

No ☐

Do you have frequent contact with animals?

Yes ☐ Which ones?____

No ☐

Diet:

☐ Normal/Diverse ☐ Vegetarian ☐ Lacto-ovo vegetarian

Other ____________________
